# Student Interprofessional Facilitator Training (SIFT) program: building capacity in clinical education leadership

**DOI:** 10.1186/s12909-022-03725-9

**Published:** 2022-09-07

**Authors:** Christie van Diggele, Stuart Lane, Chris Roberts

**Affiliations:** 1 Faculty of Medicine and Health, The University of Sydney, Sydney, NSW Australia; 2 Faculty of Medicine and Health, Sydney Medical School, The University of Sydney, Sydney, NSW Australia

**Keywords:** Interprofessional education, Leadership, Health professional, Teacher training, Peer assisted learning

## Abstract

**Background:**

While there are many teacher training programs for health professional students, few are interprofessional, and few integrate assessment and feedback prior to participation as peer teachers. In 2021, The Student Interprofessional Facilitator Training (SIFT) program was developed to allow senior students, already trained in peer teaching, to revise, build on, and practice their newly acquired skills in an interprofessional context. The aim of this study was to explore participant perception and performance, and the contextual factors that influence student aspirations as clinical teachers.

**Methods:**

Alumni of the 2021 Peer Teacher Training program (*n* = 74) were invited to participate in the SIFT program. Those who participated were invited to attend individual semi-structured interviews. Thematic analysis was used to code and categorise data into themes, using Communities of Practice as a conceptual framework. Skills in interprofessional facilitation were observed, assessed and students were provided with individual feedback. Assessment data were analysed using descriptive statistics.

**Results:**

Sixteen students from six disciplines joined the SIFT program, and 13/16 (81%) completed. Students were from medicine, nursing, diagnostic radiography, medical imaging, dentistry and speech pathology. Students reported an increased recognition of teaching as a learned skill, development of clinician identity formation as educators, development of interprofessional communication skills, increased awareness of the roles of other health professions, and an increased understanding of leadership. Participants expressed a desire for additional opportunities for interprofessional networking and peer teaching. A good level of competence in facilitation skills was reached by participants.

**Conclusion:**

The SIFT program provided a sustainable framework for health professional students to develop and evidence their teaching and leadership skills in an interprofessional context. This study highlighted the important role of observation, assessment and feedback in student teacher training programs. The process of clear assessment guidelines, direct observation with feedback from supervisors provided a way to ensure quality improvement in peer teaching. The SIFT program will help to build capacity of interprofessional programs where large numbers of teachers are required for small group teaching. The next step will be to ensure a variety of opportunities within interprofessional contexts, and with face-to-face engagement.

## Background

Teaching is well recognised as a core professional skill required by all health professionals, including new graduates. Early in their career, clinicians take on significant roles in teaching students and colleagues within their own disciplines, and across disciplines [1–3]. Yet, internationally, institutional funding is predominantly driven by clinical service provision and research, with less recognition for contributions to education and teaching [4]. Furthermore, the career path for clinical educators is often unclear [5]. Rather than being recognised as a learned skill, teaching expertise has traditionally been associated with clinical expertise [4]. Early development of a strong teacher identity for health professionals may enhance a student’s intention to be a long-term contributor to education, and their willingness to further invest in faculty development [6].

While teacher training programs for health professional students are aplenty, there is a paucity of programs explicitly linking theory to practice through long-term translation to the workplace, and provision of formal supervision, observation and feedback on teaching performance [7]. Recent literature suggests that providing this linkage may have associated benefits, including recognition of teaching as a learned skill, engagement in leadership roles and encouraging clinician identity formation as educators [8, 9]. Furthermore, provision of interprofessional contexts in health professional education activities has the potential to improve students’ communication, collaboration and leadership skills [10]. These skills are relevant to students’ future careers, where patient care is delivered in multidisciplinary teams [11, 12]. Evidence suggests that the culture of healthcare education environments is enhanced by the provision of sustainable, longitudinal professional development programs within communities that nurture educator characteristics and provide opportunities for engagement [13].

Similar to teacher training programs, interprofessional education (IPE), is seen as essential in preparing students for the health workforce [14]. With the majority of health professional student programs being taught in professional silos, IPE offers opportunities for students to work with other health professional students in team and collaborative settings more likened to the workplace [14, 15]. The inclusion of IPE in healthcare training has been linked to improvements in leadership, collaboration and communication of health professional teams, resulting in improved patient outcomes [15]. Students from various disciplines can contribute to IPE activities as facilitators, helping to scaffold the development of knowledge and skills, and sharing responsibility for shaping their own teaching and learning. However, there is paucity of guidance for students wanting to actively engage in IPE activities as a facilitator [14].

In 2021 the Student Interprofessional Facilitator Training (SIFT) program was developed at the University of Sydney to allow senior students, already trained in peer teaching, to revise, build on, and practice their newly acquired skills in interprofessional contexts. Unique to this program was the use of Entrustable Professional Activities (EPAs), allowing students to demonstrate their teaching and facilitation capabilities [16]. EPAs are increasingly being developed and used in implementation of competency-based workplace curricula and health professional education to target standardised levels of proficiency [17, 18]. Entrustment initially occurs during training, and is subsequently built upon as new activities evolve during a trainee’s career [18]. The EPA concept offers a framework that can be applied to the practice of teaching within health professional education as a tool to establish objective levels of performance, and the level of supervision required in teaching and facilitation [19–22].

### Theoretical lens

Learning theories provide lenses to help in the analysis of student experiences in educational activities [23]. Steinert (2014) posits situated learning as one of the most useful theoretical frameworks; based on the notion that knowledge is contextually situated and fundamentally influenced by the activity, context, and culture in which it is used [24]. As described by Lave & Wenger [25], the theoretical notion of communities of practice views learning as a social activity, with participant interaction being the key source of engagement and learning [25]. Communities of practice are characterised by three key elements [25]:

- Joint enterprise: a shared domain of interest, with a desire for proficiency
- Mutual engagement: joint activities that promote the development of learning relationships and collaboration
- Shared repertoire: the promotion of shared resources, concepts, experiences, and tools that are used and developed further through interactions.

Using the theoretical lens of communities of practice [25], the aim of this study was to pilot the newly established SIFT program, and explore participant perception and performance, as well as the contextual factors that influenced student outcomes. Specifically, our research questions were:

1. How do students perceive their experience of participating in the Student Interprofessional Facilitator Training (SIFT) program, and their future clinical teaching and leadership roles?
2. How competent are students to facilitate and teach their interprofessional peers?

## Methods

### Course design

The SIFT program was designed in a blended learning format, consisting of five online modules as described in Table 1: Module 1) Introduction to the SIFT program, 2) Interprofessional Education [14], 3) Leadership in health professions education [26], 4) Practical requirements and facilitation activity, and 5) Critical Reflective Task. A prerequisite of the SIFT program was completion of the Peer Teacher Training (PTT) program, comprising seven modules requiring active participation in planning and delivering a teaching session, teaching a skill, practicing clinical handover, and providing feedback [15].

**Table 1:** SIFT module outline

Module	Outcomes	Activities
**Module 1:** **Introduction to the SIFT program** Completed asynchronous, approx. 30 min Theoretical content and discussion board via Canvas	This module provides the opportunity for participants to: • Understand the layout and requirements of the program • Revise content delivered in the PTT program	Students meet individually with the co-ordinator to explain program requirements and individual student aims/goals
**Module 2:** **Interprofessional Education** Completed asynchronous, approx. 1 h Theoretical content and activity-based discussion board via Canvas	This module provides the opportunity for participants to: • Prepare for interprofessional learning activities • Evaluate the needs for interprofessional facilitation • Consider the benefits and challenges of implementing and facilitating interprofessional activities	Provision of theory and online discussion boards • Activity 1 (discussion board): Facilitating interprofessional education
**Module 3:** **Leadership in health professions education** Completed asynchronously, approx. 1 h Theoretical content and activity-based discussion board via Canvas	This module provides the opportunity for participants to: • Identify the differences between leadership and management • Describe the meaning and difference between ‘transactional’ and ‘transformational’ leadership • Describe the concept of ‘distributed leadership’ and the roles within successful teams • Evaluate key leadership competencies for health professional educators • Recognise the challenges health education leaders may encounter	Provision of theory and online discussion boards • Activity 1 (discussion board): Characteristics of leadership • Activity 2 (discussion board): Organisational leadership
**Module 4:** **Practical requirements and facilitation activity** Delivered synchronously with individual students and the coordinator via a meeting and email communication. Approx 20 min Followed by the synchronous practical assessment and feedback session. Approx. 2 h	This module provides the opportunity for participants to: • Identify the interprofessional learning session of interest for facilitation • Facilitate a small group teaching session (with supervision and feedback) • Discuss requirements with coordinator and complete any preparatory material or training	• Meet with the SIFT coordinator and select an activity. The coordinator explains the facilitation requirements and discusses any training needs related to the selected activity • Students complete the practical facilitation requirements e.g. small group teaching of the Peer Teacher Training program • Students facilitate and receive verbal feedback from their observer • Observer completes EPA rubric based on teaching performance and provides verbal feedback to participants at the end of the session
**Module 5:** **Critical reflective task** Completed asynchronously with reflective task submitted via Canvas assignments Followed by a meeting with coordinator and student for feedback and setting future goals Approx 1 h	This module provides the opportunity for participants to: • Reflect on their learning, facilitation experience, and future teaching goals • Gain personalised feedback on their facilitation session and written task	• Students complete a 500-word critical reflective essay based on their facilitation experience. Key concepts covered include (identification, subjective, objective, assessment, future planning comments) • Students attend a verbal feedback session with the co-ordinator to review their reflection and EPA rubric feedback from practical session, and discuss future goals and teaching opportunities available to them

Although a range of practical peer teaching activities could have been used for the practical component of the SIFT program (module 4), choices were restricted due to the disruptions in curriculum delivery created by COVID-19. For this study, SIFT participants co-facilitated the Peer Teacher Training (PTT) program online to fulfil the practical teaching requirements. Each SIFT student was required to facilitate a small group of senior health professional students (4–5 students) who were completing the PTT program. The duration of the activity was approximately 1.5 h. The PTT students taught a five-minute healthcare topic, and provided feedback to their peers within the small group. As the facilitator for the small group, the SIFT student was required to manage the group, lead the introductions, time each activity, and provide feedback to each student on their teaching. Each SIFT student was observed in their facilitator role, by an experienced clinical educator or academic, assessed and provided with feedback.

### Supervisors

An interprofessional team of 13 facilitators including two educationalists, and 11 clinicians from medicine (*n* = 8), nursing (*n* = 2), and physiotherapy (*n* = 1) provided observation, assessment, written and verbal feedback to each SIFT student.

### Certificate of completion

After successful completion of the SIFT program, students received a certificate to evidence successful completion of all modules, and competency in facilitation skills.

### Study design

#### Recruitment

All alumni of the Peer Teacher Training (PTT) program held in February 2021 (*n* = 74), including students from medicine, pharmacy, health sciences, nursing and dentistry were invited by email to take part in the SIFT program.

### Data collection and analysis

#### Semi-structured interviews

All participating SIFT students (*n* = 13) were invited to attend individual semi-structured, in depth interviews via Zoom, of which, 12 (92%) participated. Of the participants, 6 were male and 6 were female. The interviews ranged from 30 to 45 min in duration, and were conducted by the first author (CvD) using a semi-structured interview guide, designed to explore students’ perceptions and experience of the SIFT program. Participants were asked to describe their experience of the SIFT program, making recommendations for future improvement. They were also asked to describe current styles of leadership and interprofessionalism they have experienced in their clinical placements, and to reflect on future ways of working, having completed the SIFT program. The interview data were transcribed verbatim. After immersing ourselves in the data, and reflecting on our own field of practice, the three authors used framework analysis to code a portion of the dataset. This was done independently, using Communities of Practice [25] as a theoretical framework to identify recurrent themes and subthemes for interpretation [27]. Once meaning and any difference in interpretation of the data had been negotiated between the authors, the first author then applied the coding framework to the complete dataset [27].

### Assessment of knowledge and skills

Skills in facilitation were formatively assessed based on competencies demonstrated during a small group session of the Peer Teacher Training program. Using a prepared marking rubric and written comments, formative assessment sheets were completed by the supervisor (clinician or academic facilitator) on each of the SIFT students and submitted online via Qualtrics. Immediately following the activities, individual feedback was provided to each SIFT student. Competencies were framed as EPAs, established for each facilitation competency and provided to SIFT students and supervisors prior to the activity. The EPA descriptors included:

- Evidence of prior planning and organisation
- Demonstrated an ability to communicate clearly with students
- Demonstrated an ability to facilitate team discussion
- Provided the learner with guidance on the activity as required
- Showed consideration for multiple viewpoints of different healthcare students
- Provided learners with the opportunity to ask questions
- Supported and encouraged team interaction and involvement
- Responses to questions were appropriate, with referrals to senior staff where required

Using EPA ratings adapted from Rekman et al., 2016, each student was rated in one of the four categories based on their performance [28]:

1. “I needed to facilitate”. The SIFT student required a lot of guidance or was unprepared for the session.
2. “I talked them through it”. The SIFT student was able to perform some tasks but required repeated directions.
3. “I had little input”. The SIFT student demonstrated independence and only required intermittent prompting.
4. “I only supervised”. The SIFT student functioned independently and only needed assistance with nuances or complex situations.

Descriptive statistics were used to analyse quantitative data. Thematic analysis of the comments provided by the supervisors was performed by all authors (CvD, CR, SL) to determine prevalent themes [27].

### Ethics approval

The University of Sydney Human Research Ethics Committee approved the study, protocol number 2021/057.

## Results

In total, 16/74 (22%) of the available pool of students registered for the SIFT program in 2021, and 13/16 (81%) of these students completed the program. Students were from 6 disciplines: Medicine (*n* = 6, 46%), Nursing (*n* = 2, 15%), Diagnostic Radiography (*n* = 2, 15%), Medical Imaging (*n* = 1, 8%), Dentistry (*n* = 1, 8%), and Speech Pathology (*n* = 1, 8%).

### Semi-structured Interviews

In total, 12/13 (92%) of students attended the individual semi-structured interviews. Of these participants, 6 were male and 6 were female. Extracts from the student interview data are presented in Tables 2, 3 and 4 to answer the first research question, “How do students perceive their experience of participating in the Student Interprofessional Facilitator Training (SIFT) program, and their future clinical teaching and leadership roles?”.

**Table 2:** Themes relating to “Joint Enterprise” (a shared domain of interest and a desire for proficiency)

Themes relating to “Joint Enterprise”	Examples of comments provided by students
**Students appreciated the provision of theory and preparation provided prior to the actual facilitation activity**	*I really enjoyed it… …I liked how there were learning modules before we actually took part in a practical session. (Dentistry student)* *I also found Module 2 on leadership really interesting, about transactional and transformational leadership. This knowledge will definitely shape how I view my experiences in future clinical settings and how I behave as a future clinical professional. (Medical student)*
**The modules were clear and the workload was manageable. It could be worked through asynchronously without interrupting clinical placements**	I *found that it was really easy to follow. So, you have some clear modules, you had clear things that you needed to do and I liked that you could work through them at your own pace. (Radiography student)* *The program was really good. I liked that it is self-directed, that you can do it at your own pace. That was really helpful, especially when it is alongside the Masters course, which is quite intense. So it is good the onus is on us to do our own learning at our own pace, but the end goal and expectations are still there… (Nursing student)* *A good thing about it is that with my requirements to study in medicine and to cover a lot of theory on a daily basis and study, I didn’t feel the program was content heavy, it was very doable, in a good amount of hours and it gave me a foundation for me to build on.. …without interrupting my education, or my daily number of hours of studying, or my placement. It was very doable. (Medical student) *
**Students felt it was valuable to have a way to formally engage in teaching & teacher training**	*I want to be more involved in teaching as I go through the healthcare system – and it is one thing you never get taught. They always say there are two things you are never taught. You will become a manger, but you are never taught how to manage people and as a doctor, and you are never really taught how to teach people. The same complaints you get from lecturers and faculty – you are rewarded for research, not for teaching. (Medical student)* *It is nice to have something to formally engage in teaching. (Medical student)*
**Students valued developing feedback skills**	*The thing that really interests me the most is about giving feedback. That part was covered during the modules but also helped me to apply that knowledge or apply that theory in the practical sessions… which develop these techniques of what works for me in giving feedback, the certain words to use, the way a phrase might be taken on my reflection to others, it just it helps me perfect that small skill, but it has a huge impact for my experience or for my career at least*. *(Medical student)*
**Provided an opportunity to develop leadership skills, and consider themselves as role models and reflect on how they will practice in the future**	*I found the overall SIFT program very informative… it gave me an opportunity to really develop all of my leadership skills, but also it had a practical component that helped me facilitate that. (Speech pathology student)* *With the leadership module…. the literature and also the discussion threads on people’s experiences with transformational and transactional leadership…that was really interesting…learning about that definitely makes me reflect on how I would want to practice as a clinician in my career. And my relationships with other people, how like my actions can have a positive impact on other people in terms of inspiring them and being a role model. Role modelling is really important as you progress through your career because in health care, you’re always training younger professionals. That’s how they learn and how you learn at a point and so in that sense transformational leadership is good to know about so that when you’re at that position you can be better to the next generation. (Radiography student)*
**Students were motivated to participate in the program because they had a desire to improve their skills in teaching based on their own learning experiences**	*What made me interested in this program specifically is that I spent a lot of time in clinical placements and engaging with mentors and supervisors and also engaging with students in the more junior years and I realised how important teaching and giving feedback is and how it can make a huge difference in someone’s experience on a daily basis. I saw the SIFT program as a great opportunity not only for me right now but also for the future years, just like the little moments of giving feedback, on the job on a daily basis, I thought this program would be extremely helpful to that and so far, I’ve already seen good results*. *(Medical student)*
**Peer teaching was considered a valuable method to improve one’s own knowledge and skills**	*I will be teaching in the future. Peer teaching is so useful. You can’t teach something if you don’t know it yourself – just like studying for an exam, you are learning off each other. (Nursing student)* *…a valuable opportunity that was well run and I received valuable insight into my performance. (Medical student)*

**Table 3:** Themes relating to "Mutual Engagement" (joint activities that promote collaboration and the development of learning relationship)

Themes relating to "Mutual Engagement"	Examples of comments provided by students
**Highlighted the importance of learning interprofessional skills early on, and relevance for future career**	*The peer teaching training program was really useful… this was a natural step up from that—as you do these programs you kind of realise the significance of interprofessional collaboration and education. This program was useful… as you progress more in your career these sorts of skills are really useful to have. And if you’re good at it from when you’re a young clinician, that will make you a better clinician as you progress through your career. (Radiography student)*
**Valued the discussion boards within the modules to gain an understanding of the experience of others, and reflect on their own**	*I really liked how there was all of the discussion boards. It forced me to really reflect on my own experiences and also look at other people’s experiences, and learn from them as well. (Speech pathology student)*
**Students would appreciate future networking and teaching opportunities, including face-to-face opportunities**	*I definitely enjoyed this program so if there are other opportunities that arise I’d definitely love to partake in them and meet new people. In this program and the PTT there was always interesting people and their discipline. (Radiography student)* *It makes those events more fun when you meet someone new and they’re from an interesting discipline. Networking in terms of.. you met people within your own profession, that would also be important networking and it would be more important the further up you are in your career. (Radiography student)* *It would be so much better if it was actually face-to-face and there was a bit more collaboration. Hopefully after the lockdown we will have the opportunity to do that. (Speech pathology student)*
**While some students felt comfortable teaching within the PTT as they were familiar with it, others would prefer a variety of teaching experiences**	*I wish that for the practical component we were given different situations. Just because I took part in the Peer teacher training program already, and I just felt that when I was facilitating it, yes it was from a different angle, but I have already experienced it. So, I was just hoping if there were more opportunities in the future to facilitate activities in other settings. (Dentistry student)* *It would have been great if we had more practical experiences because I really enjoyed putting all of the theory into practical knowledge. And because it was the first time that I did facilitate a training session, I would love to do more in the future just to develop all of my skills. (Speech pathology student)*
**Students felt the interprofessional aspect was relevant to their future work practice**	*The SIFT program was super relevant because I’m a nursing student so almost everything I do is going to be interprofessional. (Nursing student)* *I feel like it was really relevant, not only to speech pathology but also across all other disciplines because it really teaches you how to work interprofessionally which is such an important skill out in the workforce, and we don’t actually have anything in our separate disciplines that actually teaches that to you. So I’m really happy that I actually enrolled in this. (Speech pathology student)*

**Table 4:** Themes relating to "Shared Repertoire" (the promotion of shared language, resources, concepts, experiences, and tools used and developed through interactions)

Themes relating to "Shared Repertoire"	Examples of comments provided by students
**Students appreciated being able to use the structured frameworks provided**	*It’s just giving us a structured framework…towards what we should actually do when we are facilitating a group, especially one with people from different health backgrounds. (Dentistry student)* *The structure of facilitating is really good in high stress or time poor situations to have a structure to go back to is really useful*. *(Nursing student)*
**Students valued the briefing session provided immediately before the teaching session**	*We had our five minute briefing session before the session that it was really clear what was going on and what we were required to do. I think it was lucky that I was able to facilitate the PTT program, so that is something that I did last year, so I was familiar with it as well. (Nursing student)*
**Interprofessional aspects highlighted the importance of understanding the knowledge and role of different health professions, and learning how to interact and foster teamwork**	*Once we are in the workforce we need to deal with people from different health backgrounds everyday so it’s really important that we understand that. You know I’m from a dentistry background and not everyone thinks that teeth are important. There are nurses and doctors, they may prioritise other things and it’s important to not only know how the knowledge of different fields are different, but also….the way that you interact with people and get that point across. (Dentistry student)* *How to foster better teamwork, between people of different disciplines I think is quite important. (Dentistry student)*
**Participants appreciated learning different perspectives of healthcare from others, which mirrors workplace experiences**	*We have different people from different specialities discussing health care topic—the exact same thing that we have in actual hospital placements where we have multiple different people joining into a meeting—coming in with different perspectives, about healthcare topics. It’s very important to see where everyone is coming from and be open minded to not always think that my perspective or my opinion is always right. That sort of helped me to pause and realise this and make sure that I apply this on the job itself. Just taking these few minutes to actually listen to other people with different opinions and different perspectives on maybe a topic that I thought I would understand really well, but seeing somebody else’s perspective adds to your experience and your knowledge. I definitely think it’s quite reflective of the actual experience in real life working with other specialities. (Medical student)*
**Students appreciated receiving a certificate that demonstrates interprofessional involvement and development of leadership and facilitation skills**	*The certificate was definitely useful because I can include it in my portfolio and also for future job prospects, I can demonstrate that I was involved in a program where I was working interprofessionally and I know all about leadership and how to facilitate good leadership. (Speech pathology student)* *I will use the certificate in the future. I want to keep doing SIFT and make sure that whoever I am teaching gets the most out of it. And to have the certification because it is quite difficult to show people in an interview in a hospital. The supervisor needs to be able to judge that you have been engaged in improving your teaching skills rather than someone who just wants to. (Medical student)* *What I gain from the program will be helpful in my career, but my certificate is something that I can also talk about in future interviews or in future job applications. Having these skills will definitely add to my profile when I’m applying for future jobs. I’m sure employees are looking for someone with these skills in the future. (Medical student)* *I will be starting an intern job and part of the duties of an intern is to teach. So having those skills will be very important on the job. (Medical student)*

Table 2 illustrates the sub-theme of joint enterprise. Participants found it valuable to have a formal way to engage in teaching, with those with a mutual interest. They found the preparation material clear with a manageable workload, particularly since it could be completed asynchronously. They felt the course provided opportunities to develop proficiency in leadership skills relevant to their future roles as peer teachers at university and clinical educators in the workforce.

Table 3 illustrates the subtheme of mutual engagement. Students highlighted the importance of learning interprofessional skills relevant to their future careers. They valued engaging in the online discussion boards to gain an understanding of the roles of other health professionals. They would appreciate future opportunities to network, collaborate and again put theory into practice in an interprofessional context, ideally in a face-to-face setting.

Table 4 illustrates the subtheme of shared repertoire. Students found the structured framework gave them the tools needed for teaching in the workplace across disciplines. They emphasised the need to develop an understanding of how other health professions communicate. Students felt the experience provided in the small group sessions mirrored experiences found in the workplace, where multiple professions meet to discuss patients/clients. The certificate provided was viewed as useful in demonstrating competency in leadership, interprofessional communication and teaching skills when seeking career advancement and as part of their degree portfolios.

### Assessment of student competencies

In total, 12 of the 13 SIFT participants consented to their formative assessment data being used in the study. In order to address our second research question, “How competent are students to facilitate and teach their interprofessional peers?”, the rating of students across each of the EPA descriptors were analysed. Students displayed a reasonable level of competence in most competencies, with the supervisor required to ‘have little input’ or ‘only supervise’. Within the competency of “Responses to questions were appropriate with referrals to senior staff where required”, one student needed guidance from the supervisor (‘I talked them through it’).

Qualitative feedback provided to SIFT students by the small group facilitators (clinical and academic staff) displayed a good balance of positive feedback and suggestions for improvement. Positive feedback emphasised the provision of clear instructions and introductions at the start of the session; the creation of a supportive and friendly environment; adaptability; adherence to timeframes; provision of feedback and appropriate use of questioning; and provision of a clear summary and conclusion to the session. Areas for suggested improvement included taking more time for introductions; the need to listen to students as a facilitator, rather than dominating peer student presentations; provision of a clear plan; and time management skills.

## Discussion

We sought to explore participant perceptions of a new Student Interprofessional Facilitator Training (SIFT) program, the level of competency developed, and the contextual factors that influenced student learning outcomes. Our findings suggest that the process of engagement throughout the SIFT program had many associated benefits. These included: increased recognition of teaching as a learned skill, development of clinician identity formation as educators, development of interprofessional communication skills, increased awareness of the roles of other health professions, and an increased understanding of leadership. Participants expressed a desire for additional opportunities for interprofessional networking and peer teaching. A good level of competence in facilitation skills was reached by participants. Our findings, however, highlight the importance of opportunities for practice, direct observation, formative assessment and individualised feedback. These findings are further explored in relation to existing literature using the conceptual lens of communities of practice.

### Joint enterprise

The SIFT program could be viewed as a joint enterprise in formally preparing students as peer teachers and future clinical educators. The SIFT program provided a shared domain of interest, facilitated by the structured, modular, self-directed format, with clear instructions, and an appropriate amount of content. This allowed students to work through the modules asynchronously, alongside their busy health curricula schedules. Small group activities assist in creating a process where shared decision-making is fostered by students interacting, collaborating and listening to each other [29, 30]. This also helps communication to become more open and collaborative, with an appreciation of the diversity of knowledge within the group [31].

Notably, students felt completion of the leadership module helped them to identify and increase their awareness of leadership styles they currently witness in the workplace, and characteristics they would like to emulate in their future careers as role models. Although rarely taught within undergraduate health professional training, effective leadership in healthcare education is increasingly recognised as essential to achieving high standards of education, research and clinical practice [32, 33]. Students valued the opportunity to develop their knowledge and skills in teaching. However, they recognised that teaching is commonly viewed as not being rewarded in the health professional workforce. It is important that institutions foster students’ desire for lifelong learning, where teaching skills are continually refined, and that systems exist to shape a teaching culture [34, 35]

Encouragingly, student comments indicate that their experience was enhanced through the use of EPAs, and a clear rubric that defined the requirements to demonstrate competency. They appreciated the targeted feedback, with a dedicated time for provision of both written and verbal feedback from supervisors. This is in line with recent literature demonstrating that the use of EPAs in the clinical setting has been shown to enhance student feedback when delivered by a trustworthy supervisor in a safe environment, immediately following an observed activity, and highlighting both strengths and points for improvement [18, 36]. While assessment results demonstrate that students were reasonably competent to facilitate small group sessions with their peers, this also emphasises the need for adequate supervision, assessment, and feedback while students develop their teaching capabilities, prior to carrying out unsupervised teaching tasks.

### Mutual engagement

Our findings highlight the need for opportunities for students to practice facilitation on a variety of topics within interprofessional contexts to develop relevant practical teaching experience. This is supported by evidence that in order to reinforce learned skills, multiple opportunities for practice need to be made available [37]. Students appreciated the flexibility that the online SIFT program brought but expressed a preference for face-to-face opportunities. While online activities have the capacity to increase flexibility, greater enjoyment and increased development of skills is known to occur through face-to-face activities [37, 38]. Furthermore, it is well recognised that face-to-face interactions play an important role in developing relationships with peers and teachers and networking opportunities [39]. Certainly, students expressed a desire to network and collaborate as alumni of the SIFT program. A willingness of network members to share their knowledge is key to success of interprofessional programs [40]. Social relationships help support development of health professionals’ identity as teachers [5], and it will be important to build on this network.

Motivation, institutional support and effective networks all contribute to the development of professional identity [13]. Students mentioned altruistic reasons for participating in the SIFT program, such as helping with the sustainability of the PTT and other interprofessional programs, and to help ensure the quality of teaching for other students. The certificate of completion was also identified as important by students, as it was regarded as a valuable means to enrich their portfolio for future job applications. That is, evidencing not only their commitment to training in teaching, but also demonstrating interprofessional and leadership skills. For some students, it was their own good and poor experiences as learners that motivated them to participate in the SIFT program. This teaching activity also helped to reinforce knowledge of their own healthcare learning topics, and motivated them to review topics. In situated learning, the context has a strong influence on what learning is taking place through interaction, co-participation and interaction with others. As noted by Sargeant (2009), both of these elements are also essential elements of IPE [41]. Building on this, the community of practice created through the SIFT program, with individual preparation and small group activities, helped students to work together, and collaboratively create their new knowledge and skills [41].

### Shared repertoire

Literature suggests that early experience of IPE helps to enhance students’ readiness for further interprofessional learning and their attitudes to multidisciplinary teamwork [42, 43]. Through interprofessional activities conducted in the SIFT program, students shared their disciplinary knowledge with each other, with the content being simultaneously relevant to learning needs across disciplines. The activities were viewed by students as mirroring activities that occur in the workplace, such as interdisciplinary meetings. However, it is possible that the interprofessional value of the activity was reduced due to the lack of face-to-face interaction [40]. Participants appreciated facilitation by various health professionals, helping to build social capital across disciplines [44]. The presence of role models, mentors, the academic environment and training provided all contribute to the development of professional identity [13, 45–48].

Students recognised the importance of interprofessional activities and teaching skills development during their university education. They felt this was reinforced with the theory provided in the modules, as well as the opportunities for practice as facilitators. The learning frameworks and models provided common tools that allowed students to feel well prepared before practice. The practical activities helped to foster their skills in teamwork and communication with other disciplines—skills that are not otherwise explicitly taught in their healthcare curricula. Achievement of an education-focused health professional workforce is reliant on long-term faculty development programs that provide informal experiences in group settings, and are well supported by institutes [9] Although there are many challenges to conducting interprofessional learning activities, it provides a promising pedagogical tool for preparing students for collaborative practice in the workplace [49].

### Limitations

Our study is the first of its kind to explore the professional development pathways of student interprofessional educators. Although the SIFT program participants were recruited on a voluntary basis, the 12 interviews had sufficient information and depth to demonstrate the uniqueness of their views, through their shared experience of the SIFT program). With these caveats, our results may be of value to interprofessional educators in other contexts and settings seeking to adapt the principles to provide their own pathways to capacity building in student interprofessional clinical teaching. This study also highlighted the important role of observation, assessment and feedback in student educator training programs.

## Conclusion

Our findings indicate that the SIFT program provided a sustainable framework for health professional students to develop and evidence their teaching and leadership skills in an interprofessional context. This study also highlighted the important role of observation, assessment and feedback in student teacher training programs. The process of clear assessment guidelines, direct observation with feedback from trusted supervisors provided a way to ensure the quality improvement of peer teaching and skill development that can be taken into the workforce. The SIFT program not only provides opportunities for health professional students to develop skills in leadership and interprofessional facilitation, but in addition, will help to build capacity of interprofessional programs where large numbers of teachers are required for small group teaching. The next step will be to ensure a variety of opportunities in various interprofessional contexts for both SIFT alumni and newcomers, and to renew face-to-face engagement post-Covid.

## Data Availability

Datasets supporting the conclusions of this article are included within the article. Additional data at the level of individual participant is not available as per confidentiality agreements approved by the Human Research Ethics Committee, University of Sydney.

## Abbreviations

EPA: Entrustable Professional Activity
IPE: Interprofessional Education
PTT: Peer Teacher Training
SIFT: Student Interprofessional Facilitator Training
